# Updates in pelvic neuromodulation: the role of pelvic neuromodulation in pelvic disorders

**DOI:** 10.3389/fruro.2024.1329305

**Published:** 2024-03-15

**Authors:** Baydaa Alsannan, Mai Banakhar, Magdy Hassouna

**Affiliations:** ^1^ Department of OBGYN, Faculty of Medicine, Kuwait University, Kuwait City, Kuwait; ^2^ Faculty of Medicine, King Abdulaziz University, Jeddah, Saudi Arabia; ^3^ Temerty Faculty of Medicine, Institute of Medical Science, University of Toronto, Toronto, ON, Canada

**Keywords:** sacral neuromodulation, pelvic neuromodulation, animal experiments, chronic pelvic pain syndrome, sexual dysfunction, neurogenic bladder, neuromodulation in children

## Abstract

Pelvic disorders affecting both male and female patients are major areas of concern for clinicians in cases where pharmacotherapy and behavioral therapy are not effective. In such cases, pelvic neuromodulation has become an alternative therapy that could relieve chronic pelvic pain and enhance the quality of life. The goal of this paper was to present a summary of the current therapeutic applications of various pelvic neuromodulation techniques and their efficacy in treating patients with a range of pelvic illnesses. Based on the available literature, this review assessed the validity and significance of the last 10 years’ advancements in the fields of sacral neuromodulation (SNM), posterior tibial nerve stimulation (PTNS), and pudendal neuromodulation (PNM), including meta-analyses, randomized controlled trials, and observational, prospective, and retrospective studies.

## 1 Introduction

Three neural systems—somatosensory, sympathetic, and parasympathetic—innervate the pelvis and lower bladder. By activating the bladder’s detrusor muscle and inhibiting the urethral sphincteric mechanism, the parasympathetic nervous system, *via* the pelvic nerve (S2–S4), promotes voiding. From T12 to L1, the sympathetic contributions suppress the bladder and stimulate the urethra, encouraging storage. The pelvic floor muscles and the urethra are regulated by somatic innervation. This comes in *via* the pudendal nerve (S2–S4) (1).

The main nerve supply of the levator ani muscle (LAM) comes from S3 and S4, with the help of S2 and the coccygeal plexus. The pudendal plexus contains the levator ani nerve, which arises from S4. These muscles also receive some fibers from the coccygeal plexus and the inferior rectal nerve, a branch of the pudendal nerve. The muscles lack bilateral innervations (2).

For individuals with several disorders of the lower urinary tract, neuromodulation has emerged as a viable alternative therapy. The most often utilized and recommended neuromodulation methods in clinical practice are sacral neuromodulation (SNM), posterior tibial nerve stimulation (PTNS), and pudendal neuromodulation (PNM). Regarding the mode of action of neuromodulation, several theories exist. All three contribute modifications to the central and peripheral nervous systems, despite the fact that SNM, PTNS, and PNM exhibit their activity through distinct nerve roots (3).

One of the diverse areas of medicine with the quickest rate of growth is neuromodulation, which is used to treat thousands of patients worldwide with a wide range of illnesses (4). Neuromodulation, as defined by the International Neuromodulation Society, is a multidisciplinary field that covers science, medicine, and engineering. It involves the processes of electrical or chemical inhibition, stimulation, modification, regulation, or therapeutic alteration of activity in the central, peripheral, or autonomic nervous system (5).

**Figure 1:**
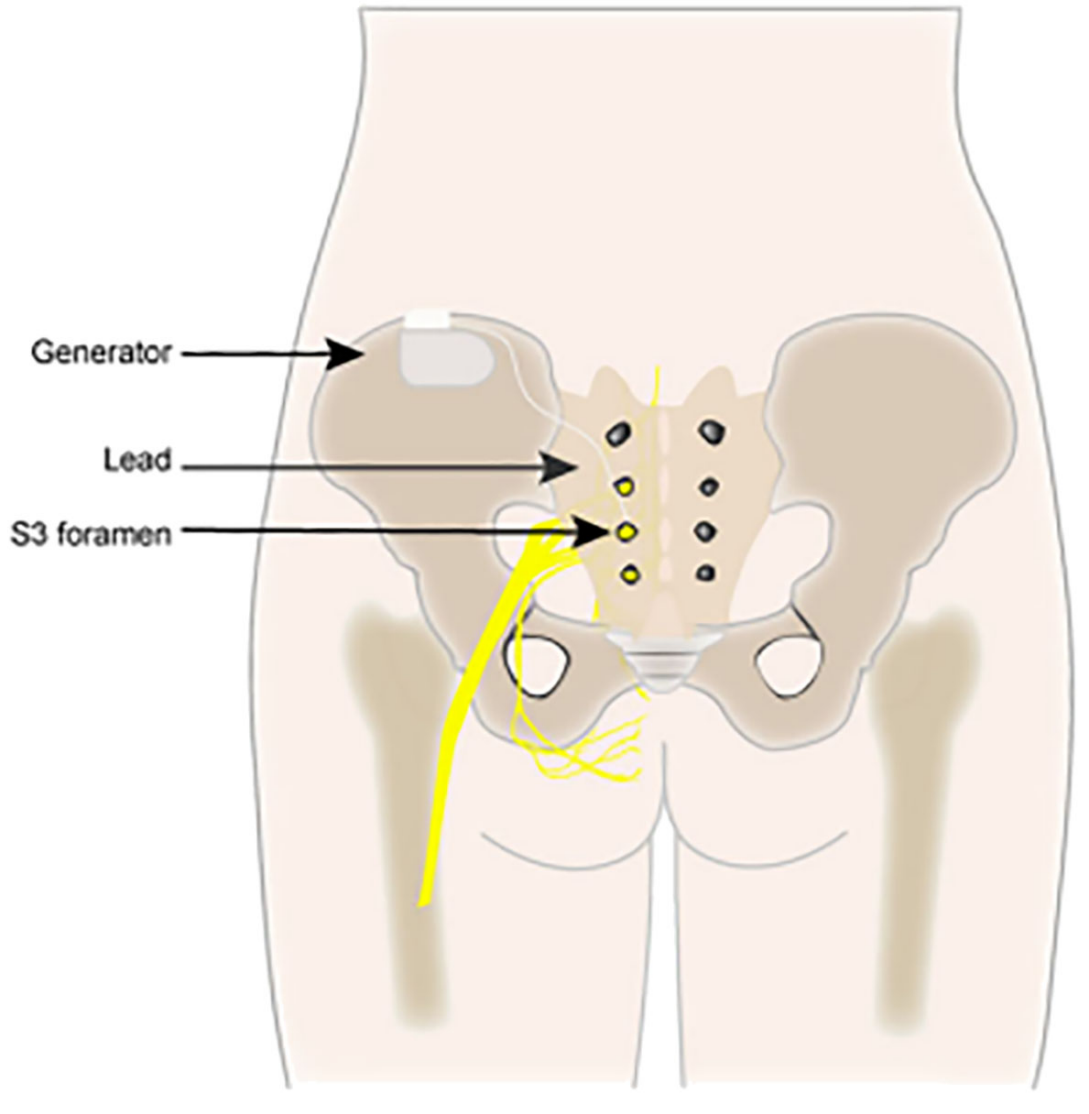
Sacral neuromodulation (SNM).

**Figure 2:**
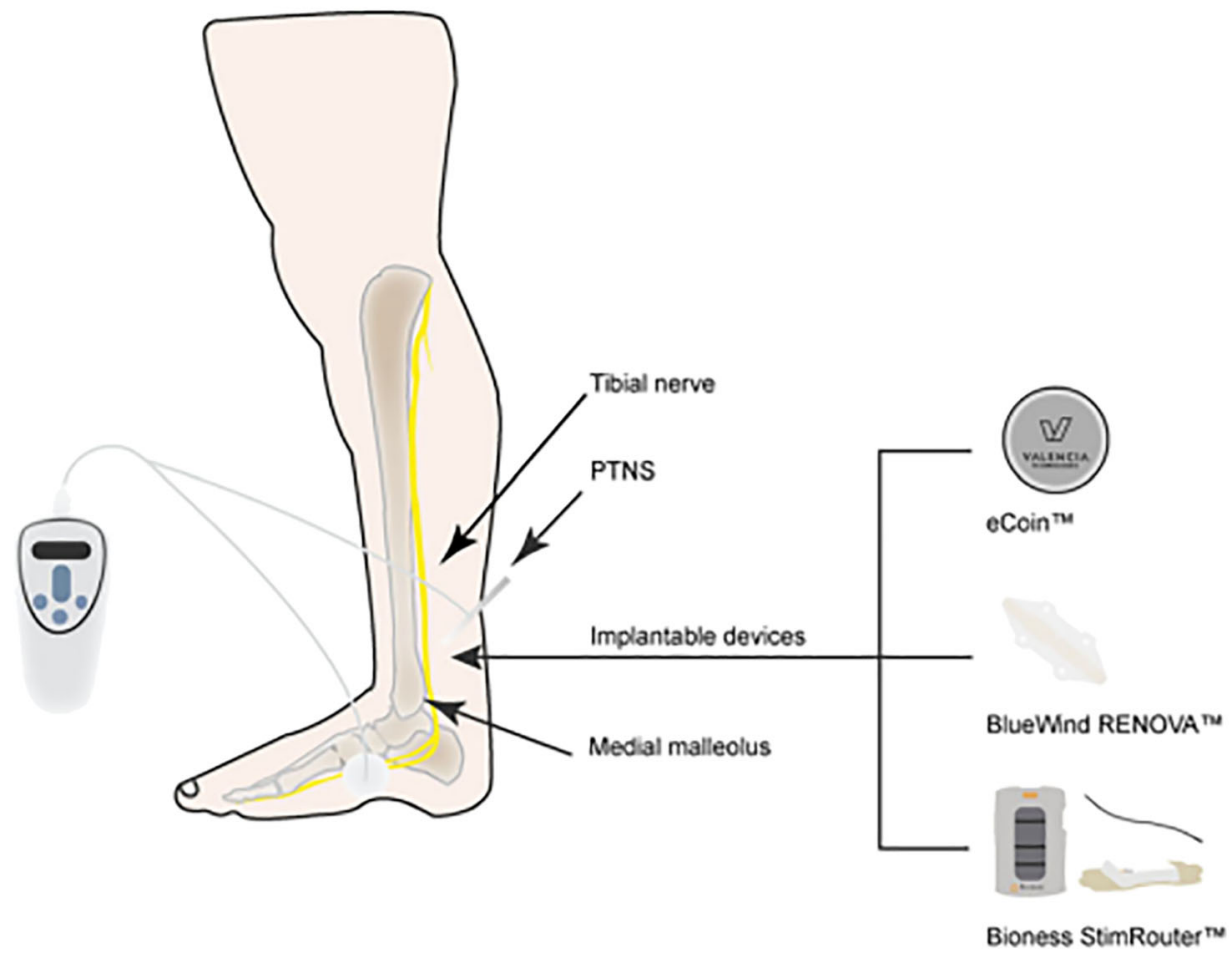
Non-implantable and implantable posterior tibial nerve stimulation (PTNS).

**Figure 3:**
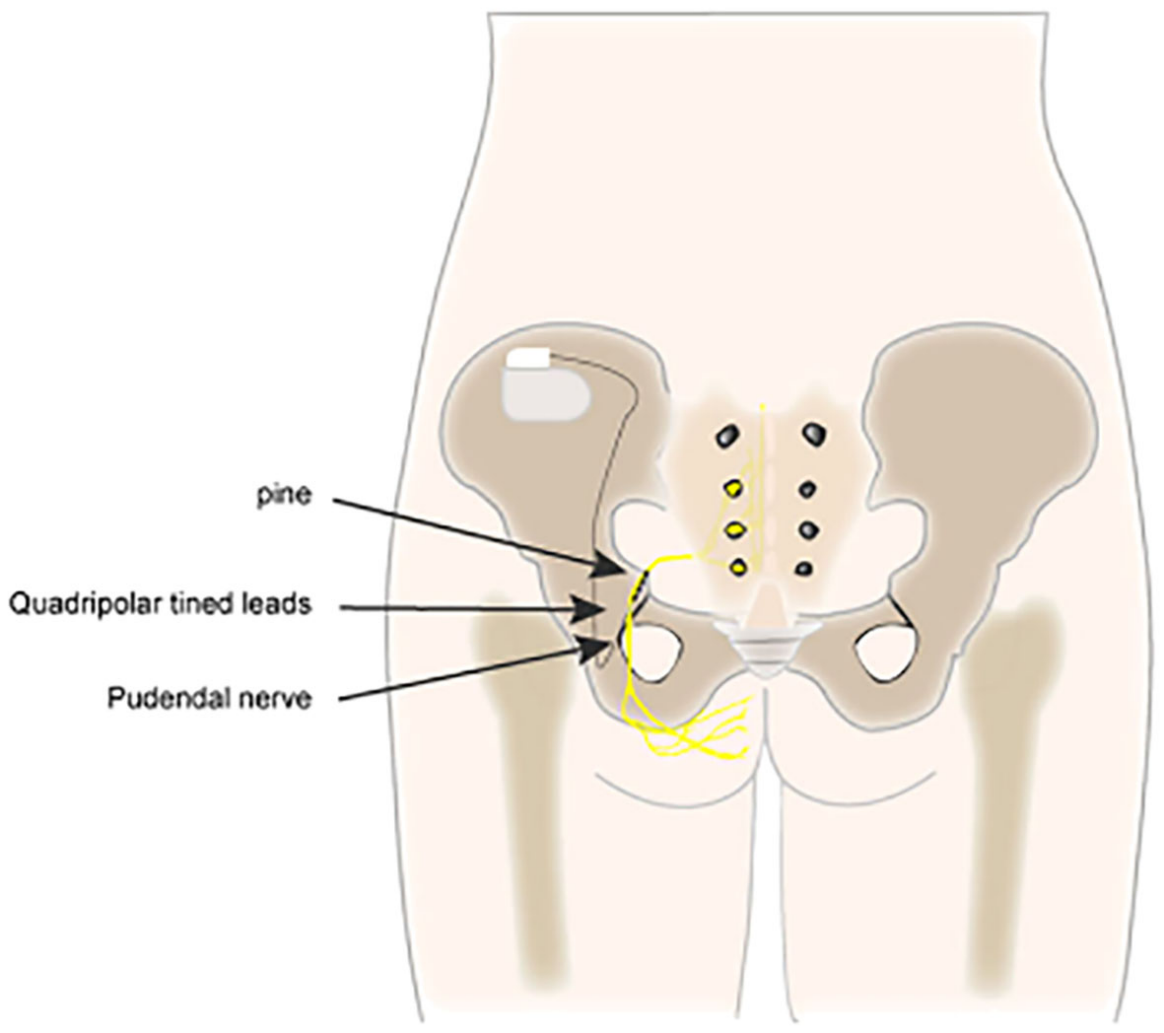
Pudendal nerve neuromodulation (PNM).

The most commonly utilized neuromodulation techniques are SNM (**Figure 1**), PTNS (**Figure 2**), and PNM (**Figure 3**) (6–8). The use of SNM has been approved by the U.S. Food and Drug Administration (FDA) for the treatment of fecal incontinence, chronic non-obstructive urinary retention, and medication refractory overactive bladder (OAB), whereas PTNS is solely approved for the treatment of OAB (9, 10). The FDA has not yet granted approval for PNM (S2–S4 nerve roots) to treat lower urinary tract dysfunction (LUTD) (10, 11). Off-label application of these methods is currently used as a therapeutic approach for a number of urinary and non-urinary pelvic floor disorders, including neurogenic lower urinary tract disorders, pudendal neuralgia, interstitial cystitis (IC)/bladder pain syndrome (BPS), chronic pelvic pain (CPP), and sexual dysfunction (12).

## 2 Methods

**Figure 4:**
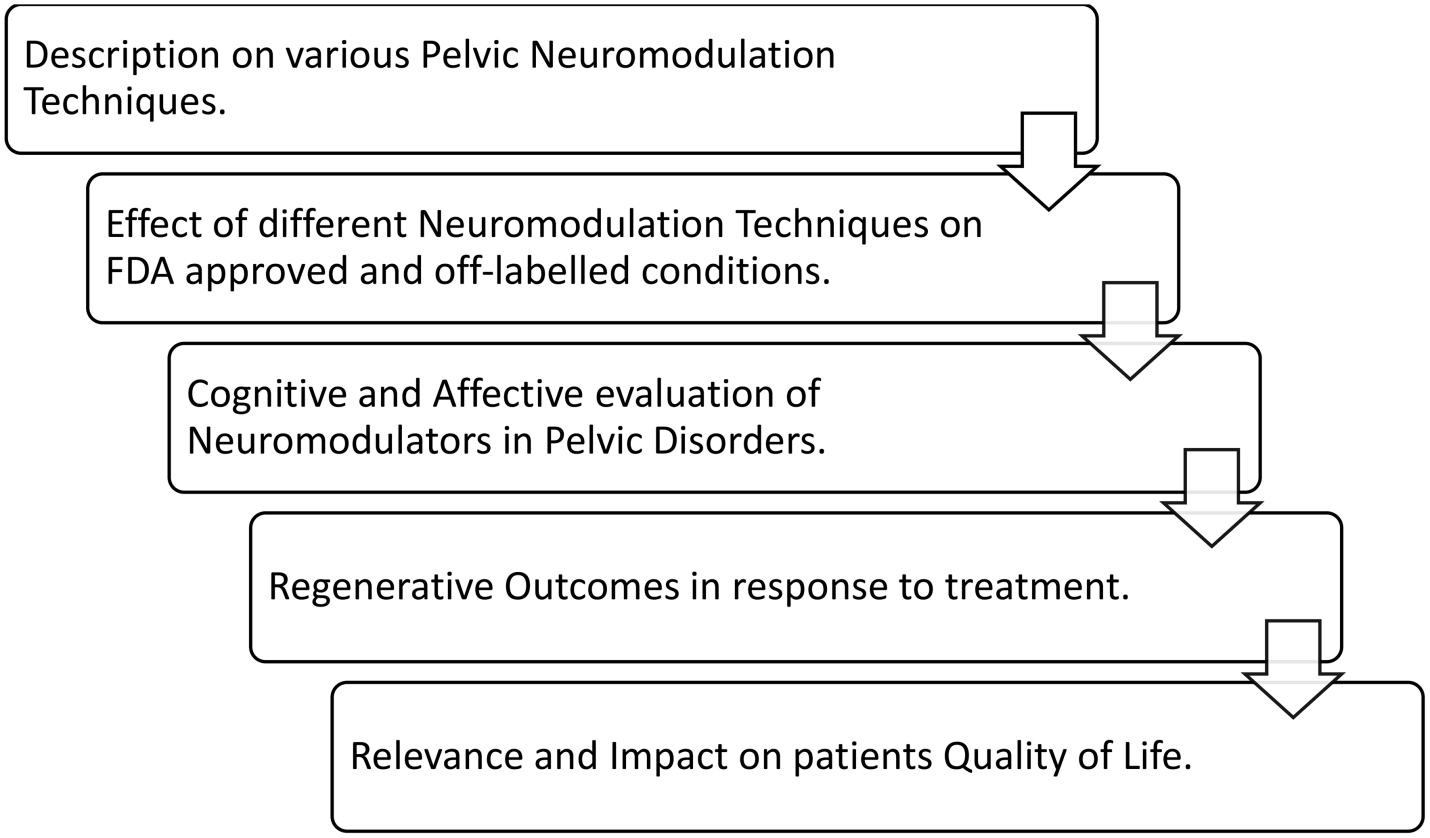
Flowchart summarizing the key points of interest in this literature review.

The available literature on the use of SNM, PTNS, and PNM for pelvic disorders was reviewed. Data were selected from literature published from 1999 to 2023, identified from PubMed, Embase, and EAU guidelines, and manual searches of known primary and review articles using the following keywords: pelvic pain disorders, OAB, neurogenic bladder, sexual dysfunction, interstitial cystitis, bladder pain syndrome, and lower urinary system disorder, the same keywords were searched in pediatric studies. We made an effort to include recent articles to a great extent. The flowchart (**Figure 4**) provides an overview of the review topics, which include information on the methods of pelvic neuromodulation, its impact on all recommended conditions, and the evaluation of subjects undergoing treatment. The outcomes, both improvements and limitations, were collated, explaining the relevance of neuromodulation techniques and their influence on the quality of life (QOL) of subjects.

## 3 Types of pelvic neuromodulation techniques

### 3.1 Sacral neuromodulation

Around 1982, Tanagho and Schmidt, at the University of California, San Francisco, carried out the first SNM surgery (13). Three chronic voiding dysfunction conditions were approved by the U.S. FDA in 1997 and 1999: intractable urge incontinence, urgency–frequency, and non-obstructive urine retention (14). Individuals who have not responded to conservative therapy or who are unable to tolerate it (15) were offered SNM therapy. The FDA authorized SNM in 2011 for individuals with chronic fecal incontinence who have failed or could not tolerate conservative treatment. In Canada and Europe, it is also indicated for chronic constipation (16). Ever since the FDA approved SNM, researchers have continuously assessed its effectiveness in treating a variety of conditions, including painful bladder syndrome, IC, neurogenic bladder, pelvic floor muscle dysfunction, CPP, sexual dysfunction, and vulvar/perineal disorders.

### 3.2 Posterior tibial nerve stimulation

The first study describing PTNS treatment for OAB in human patients was published in 1983 by Edward J. McGuire and colleagues (17). Marshall L. Stoller expanded the treatment regimens and improved the technique in the late 1990s, resulting in the 10- to 12-week protocol that is still in use today (18). PTNS was approved as a treatment method for OAB and associated symptoms of urinary frequency, urinary urgency, and urge urinary incontinence by the FDA in 2000 and the National Institute for Health and Clinical Excellence (NICE) in 2009 (19; 20). Van der Pal first described the stimulation of the tibial nerve with an implanted device in 2006 (21). PTNS uses interval (weekly) stimulation sessions of the tibial nerve at the ankle with no permanent lead, such as percutaneous and transcutaneous posterior tibial nerve stimulation or continuous implanted stimulators (BlueWind RENOVA device, Bioness StimRouter device, StimGuard LLCs, and eCoin device) (17). Patients are typically able to go home on the same day after the operation, which takes around 30 min. According to guidelines released in October 2010 by the National Institute for Health and Clinical Excellence (NICE) in the United Kingdom, “PTNS for OAB showed effectiveness without serious safety concerns” (19). The efficacy and indications of PTNS for other pelvic disorders are still unclear.

### 3.3 Pudendal nerve neuromodulation

S2 through S4 of the sacral plexus are the origins of the pudendal nerve. It travels by the greater sciatic foramen out of the pelvis, crosses the ischial spine, and then returns *via* the lesser sciatic foramen (22). The anal and urethral sphincters receive motor innervation from the pudendal nerve, while the inferior rectal branch, perineal branch, and dorsal nerve of the penis/clitoris often carry sensory information (22, 23).

Pudendal nerve stimulation is a type of neuromodulation that is used to treat urinary incontinence and pelvic pain. It is gaining interest due to its greater range of stimulation of the sacral nerve root compared to S3 alone (24). Enhancement of the function of the bladder and pelvic floor muscle groups can be achieved by stimulating the pudendal nerve, which controls the bladder and pelvic floor muscles. Numerous methods for direct pudendal nerve neuromodulation have been developed, as it might excite more sensory nerve fibers than sacral nerve stimulation and is less prone to generating side effects, such as stimulation of the leg and buttock muscles (5). The first method for stimulating or inhibiting the pudendal nerve by puncture was initially demonstrated by Schmidt in 1989 (25). Heinze et al. (26) introduced the “STAR” puncture technique, which they compared to the other three puncture procedures: the Spinelli technique (27), the Bock technique (28), and the Peters technique (29).

PNM is applied similarly to SNM, and the pulse generator is implanted only after the test stimulation reaches the standard (30). The device sends electrical pulses to the pudendal nerve, which can help improve bladder control and reduce pain. For all patients, including those who may have previously failed SNM, PNM reduces voiding symptoms by 42.8%–63% (31).

## 4 Off-label uses of pelvic neuromodulation in different pelvic floor disorders

### 4.1 Use of neuromodulation in chronic pelvic pain

Perceived to originate from the pelvis, CPP is pain that lasts longer than 6 months and is frequently linked to negative outcomes in terms of cognition, behavior, sexuality, and emotions. It can also manifest as symptoms that indicate lower urinary tract, myofascial, neurological, or gynecologic dysfunction (32, 33). The prevalence of CPP in women is notably higher than that in men, with the former having a range of 5.7%–26.6% (34). In contrast, CPP in men manifests primarily as prostatitis, with a prevalence ranging from 2.2% to 9.7% (35), and the risk increases with age (36).

The most prevalent CPPs unrelated to the reproductive system are depression, myofascial pain syndrome, pelvic floor muscle tenderness, primary urethral pain syndrome, irritable bowel syndrome (IBS), and IC, or painful bladder syndrome. These disorders have been estimated to influence 20%–60% of women who experience CPP (37–40).

#### 4.1.1 Sacral neuromodulation in CPP

A newer, minimally invasive method for the treatment of refractory CPP is SNM (41). Considering the function of the sacral nerve roots in transmitting sensory data from the pelvic floor, it is finally accepted for off-label use in resistant CPP and is a desirable therapeutic target (42–44).

Shaker and Hassouna published the first evidence of the effectiveness of SNM in BPS/IC with CPP in 1999 (45). The effectiveness of neuromodulation was observed in patients implanted for urgency/frequency syndrome and pelvic pain, which, as a result, was able to reduce both symptoms. After the failure of oral and intravesical treatments, Chai et al. reported their success with percutaneous S3 stimulation in six patients exhibiting clinical symptoms (increased voiding frequency, urgency, and pain) and cystoscopy findings (glomerulations) suggestive of IC. Subjective sensations of pain and urgency were considerably reduced (46).

A systematic review and meta-analysis published in 2022 found SNM to be effective in reducing pain and improving the QOL of patients with CPP. The mean pain score decreased by 4 to 7 points on a 10-point scale after SNM treatment (47, 48). SNM was well tolerated, with few serious side effects. The authors concluded that SNM is an effective treatment for CPP, which significantly reduces pain and increases patients’ QOL with immediate- to long-term effects (48). In addition, SNM is an effective therapy for CPP in both IC/BPS and non-IC/BPS patients, with better results in non-IC/BPS patients (49).

Maher et al. assessed 15 women with primary BPS using a percutaneous sacral nerve root stimulation test. Of these women, 73% requested to proceed with the second phase of implantation, indicating a considerable improvement in pelvic discomfort, voiding dysfunction, social functioning, and overall health-related QOL (50). In a retrospective analysis of 21 female patients with refractory BPS treated with SNM, 11 (52%) demonstrated a 50% improvement in bladder pain and voiding symptoms following test stimulation and were considered for permanent implantation. The patients’ symptoms were assessed using a Visual Analog Scale (VAS), a voiding diary, and a Urogenital Distress Inventory Short Form (UDI-6) (51). Significant gains in bladder pain and voiding parameters were observed at 1-year follow-up, and these benefits persisted at 5-year follow-up. In a retrospective study, Marinkovic et al. examined the therapeutic efficacy of SNM in 34 patients with refractory BPS over an average follow-up of 86 ± 9.8 months. They discovered significant improvements in the VAS score, pelvic pain, and Urgency/Frequency Patient Symptom (PUF) scale (52).

In a study of 21 patients with IBS, Fassov et al. assessed the effectiveness of SNM. As a result of stimulation, the number of daily bowel movements and discomfort significantly improved, whereas the IBS-specific symptom scores dropped, with borderline significance (53).

International guidelines recommend SNM as a fourth-line treatment for BPS/IC patients after failure of behavioral, oral, and intravesical pharmaceutical treatments, including hydrodistension. However, there is no high-level evidence supporting this recommendation. In its most recent update, the American Urological Association (AUA) recommended doing an SNM trial prior to considering major surgery (cystoplasty or urinary diversion with or without cystectomy) or oral cyclosporine as therapy for BPS/IC (54). In the same vein, the latest version of the *EAU Guidelines on Chronic Pelvic Pain* (2022 edition) also recommends SNM before considering more invasive surgeries (55).

Peters et al. compared the results of SNM following the traditional procedure [percutaneous nerve evaluation (PNE) followed by implantation of the permanent quadripolar lead] and the staged procedure (implantation of the permanent quadripolar lead in the first stage). Both SNM techniques improved urinary symptoms, pain, and QOL (56). SNM has also been shown to be effective in reducing other variables related to symptom reduction in refractory BPS/IC patients, such as the need for opioids (57).

Marcelissen et al. conducted a non-systematic assessment of literature published between 1990 and 2010. They found 11 studies that reported the results of SNM in refractory BPS/IC and two more publications that reported its effects in patients with persistent, nonspecific pelvic or urogenital pain. All studies produced positive results for SNM (58).

More than 500 patients were included in one randomized controlled trial (RCT), eight prospective cohort studies, and eight retrospective case series, according to a 2017 systematic review by Wang et al. The follow-up period was from 0 (test) to 86 months. Their analysis revealed significant improvements in the objective variables (e.g., daytime frequency, nocturia, 24-h micturition, and average voided volume) and the subjective variables [e.g., urgency and specific BPS/IC symptoms as assessed using the Interstitial Cystitis Symptom Index (ICSI)–Interstitial Cystitis Problem Index (ICPI) questionnaire] (59).

A long-term study, with a mean follow-up of 96 months, on SNM for patients with BPS/IC who did not respond to third-line treatment was published by Hernández et al. It was discovered that 6 out of 10 patients benefit from SNM in the mid- and long-term, with the only major side effect being a lead rupture during the test phase that needed to be extracted by open surgery (60).

#### 4.1.2 Posterior tibial nerve stimulation in CPP

Although PTNS has mostly been used to treat OAB, some studies have shown that it can also significantly relieve other forms of pelvic pain. The first report of PTNS involving patients with radiation cystitis, IC, and neurogenic and non-neurogenic OAB was published by McGuire and his team. Urinary frequency and pain were improved in four out of five participants in the BPS/IC group (61).

Van Balken et al. investigated the effect of PTNS on CPP. At 12 weeks after treatment initiation, 42% were considered to have subjective responses, 21% had objective responses (VAS reduction of >50%), and 18% had partial responses (a reduction of >25%) (62).

In an investigation by Kabay et al., 89 individuals with primary prostate pain syndrome were randomized to receive either PTNS (*n* = 45) or sham treatment (*n* = 44). After 12 sessions of PTNS, the NIH Chronic Prostatitis Symptom Index (NIH-CPSI), pain, and urgency scale scores showed statistically significant changes in the PTNS group but not in the sham group (63).

The McGill Pain Questionnaire (MPQ), the Female Sexual Function Index (FSFI), the VAS, and SF-36 scores were utilized in a randomized controlled experiment by Gokyildiz et al. to assess how PTNS treatment affected QOL and sexual life in women with CPP. According to the findings, women in the PTNS group significantly outperformed those in the control group in terms of emotional functioning, mental health, social functioning, and pain. They also had higher FSFI ratings (64). A systematic review and meta-analysis of 16 studies published in 2021 found that PTNS was effective in reducing pain in patients with CPP. The mean pain score decreased by 3.3 points on a 10-point scale after PTNS treatment. It is well tolerated, with few serious side effects. The most common side effects of PTNS are mild pain at the needle insertion site and temporary foot numbness. Vollstedt et al. showed that PTNS can be effective in reducing pain in individuals with CPP, OAB, and IC (17).

The few available studies on PTNS have yielded inconsistent outcomes, particularly in the BPS/IC population. A prospective case series of 20 women who were diagnosed with BPS/IC based on the National Institute of Diabetes and Kidney Diseases (NIDDK) criteria was published by Ragab et al. PTNS was administered to the patients for 30 min weekly for 12 weeks. The VAS was used to assess pain, as well as the O’Leary-Sant ICSI, O’Leary-Sant ICPI, and the global response assessment (GRA) score. A mere 10% of the patients at the end of the research reported a mild to moderate improvement in their symptoms (65).

More recently, Sudol et al. conducted a pilot study including 16 patients, 10 of whom finished the regimen. All of the patients had PTNS sessions for 30 min once a week for 12 weeks, provided they matched the SUFU criteria for BPS/IC. The GRA scale was modified as the primary result, whereas the VAS and ICSI/ICPI scores were the secondary outcomes. Two patients were slightly worse, two remained unchanged, four slightly improved, two significantly improved, and one patient showed a significant improvement, according to the GRA. In addition to non-statistically significant declines in the ICSI/ICPI scores, six participants reported improvements in their VAS. The authors came to the conclusion that, although there were no statistically significant gains, PTNS should be used as an off-label treatment for patients with BPS/IC (66). With positive outcomes, some researchers have assessed PTNS in conjunction with further therapy for BPS/IC. In a longer follow-up of 13 months, Baykal et al. assessed the combination of PTNS and intravesical heparin in 12 patients. The results showed durable improvements in pain levels, cystometric capacity, and frequency of voiding (67). Their encouraging findings imply that future research is necessary to fully investigate the possibility of combining PTNS with other BPS/IC therapies.

#### 4.1.3 Pudendal nerve stimulation in CPP

One potential therapeutic option for persistent pelvic discomfort is the targeted neuromodulation of the peripheral nervous system using pudendal nerve stimulation. It is interesting to note that pudendal neuralgia is frequently diagnosed based on the ability to relieve pain following a pudendal nerve block (68). Permanent generator installation generally follows a successful trial. Electrode implantation has been done using minimally invasive needle methods and neurophysiologic guidance under local anesthesia (29, 30). Five studies with 129 patients who received PNM for persistent chronic pelvic discomfort were identified in a review by Hao et al. (47). Pudendal neuralgia and IC were the most frequently found causes of pain. More than 80% pain reduction and general high patient satisfaction were found in three investigations, which showed encouraging results (69–71). The largest study is a case series including 84 patients, and more than half of the patients reported improvements in pain (30).

The effectiveness of PNM was assessed in 20 patients with CPP by Heinze et al. They discovered that the mean pain alleviation was statistically significant only when the STAR and Bock techniques were applied. After a 4-week test period, unilateral PNM did not show any statistical significance (Spinelli and Peters techniques) (26). Peter et al. concluded, in a pilot study, that chronic PNM can improve pain in patients with chronic pudendal neuralgia (30).

### 4.2 Use of neuromodulation in sexual dysfunction

Sexual dysfunction is defined as significant distress that is caused by repeated problems related to the experience, response, and pleasure of performing sex (72). Sexual desire disorder, sexual arousal disorders, orgasmic disorders, and genital pain disorders are the different categories of sexual dysfunction (73). Sexual desire disorder consists of hypoactive sexual desire disorder (73, 74). Sexual arousal disorders include erectile dysfunction (ED) and persistent genital arousal (73, 75). Orgasmic disorders include premature ejaculation, anejaculation, and female orgasmic disorder (73, 75). Genital pain disorders include dyspareunia and vaginismus (73).

A patient’s emotional health and QOL can be significantly impacted by sexual dysfunction. Due to mental and physical discomfort, as well as loss of functionality, sex life is primarily significantly damaged (76). Poor marital satisfaction is linked to decreased sexual function (77). Furthermore, less sexual activity puts a couple’s potential to conceive a child in jeopardy. A person with sexual dysfunction increases their risk of developing depression and other mood disorders, in addition to having negative effects on their sexual life (78). Pelvic neuromodulation is a well-established treatment for several urinary and bowel dysfunctions; nevertheless, its role in sexual dysfunction remains unclear.

#### 4.2.1 SNM in sexual dysfunction

In SNM, there is a significant association between functional diagnosis and the Female Sexual Function Index (FSFI) total score, as well as the FSFI-specific domains of arousal, lubrication, and satisfaction. SNM therapy significantly improved the total FSFI score; the desire and orgasm components showed significant improvement; and QOL showed significant improvement after SNM treatment in five categories (79, 80). Significant improvements in sexual function were observed in a pooled analysis of data from 11 studies, which included 573 patients prior to SNM and 438 patients following SNM. SNM appears to improve sexual function in women with pelvic floor problems, particularly bladder dysfunction (81).

In contrast, a questionnaire used by Zaer et al. and Andy et al. reported no significant change in the capability of orgasm and lubrication in women after implantation of a sacral nerve stimulator (82, 83).

The impact of neuromodulation on erectile function was evaluated across eight studies with a total of 295 patients. In two studies that assessed the ability to maintain an erection with sacral nerve stimulation, 43 out of 47 patients reported improvement in the ability to maintain an erection (84, 85). In another study using sacral nerve stimulation, the number of patients experiencing ED decreased from nine to two (86). Lombardi et al. reported a median increase in the International Index of Erectile Function-5 (IIEF-5) scores for patients in both the neurogenic and idiopathic dysfunction groups (87). There was an overall increase in the IIEF-5 scores in the two additional studies that used sacral nerve stimulation (80, 88).

Furthermore, research revealed that the general quality of “sex life” improved. Less incontinence was identified as a contributing factor in this improvement (89, 90), with decreased urgency (91), fewer urinary and bowel symptoms (92), and overall less distress (87). SNM appears to improve sexual function in women with pelvic floor problems, particularly bladder dysfunction (81).

In a systemic review by Jin et al., which included 13 studies measuring female sexual function using the FSFI, nine studies (*n* = 305) revealed that sacral nerve stimulation significantly improved dyspareunia (93). In a case report, the VAS for dyspareunia decreased after implantation of the sacral nerve stimulator (94).

On the other hand, a retrospective observational study that included 154 patients found decreases in ejaculation, orgasm, and intercourse capability in men after implantation of the sacral nerve stimulator (82).

#### 4.2.2 PTNS in sexual dysfunction

Marinello et al. evaluated PTNS and found an overall decrease in the IIEF-5 scores (95). In another prospective observational study of 45 patients who underwent PTNS, it was reported that overall sexual desire and satisfaction increased (96). In 2016, Musco et al. reported that PTNS improved sexual function in women with dry OAB. All FSFI domains showed statistically significant improvements in women with female sexual dysfunction (FSD). In addition, women without FSD at baseline reported statistically significant improvements in their sexual function based on the FSFI scores (97).

In four out of seven trials, a systematic review indicated that PTNS improved sexual function. General sexual function improved significantly with PTNS, according to a meta-analysis of four investigations. Despite the limited sample sizes, the results are promising in terms of the positive effect of PTNS on sexual function (98).

In an animal study, Zimmerman et al. successfully demonstrated the ability of tibial nerve stimulation to increase vaginal blood perfusion in anesthetized female rats and ovariectomized rats. For up to 5 weeks following ovariectomized rats, PTNS temporarily increased the vaginal blood perfusion during stimulation but had no effect on the serum estradiol levels, body weight, or blood glucose. According to this study, tibial nerve stimulation may be utilized to treat female sexual interest/arousal disorders related to genital arousal by increasing pelvic blood flow (99, 100).

The sexual behavior of menopausal female rats was examined in 2023 to determine the short- and long-term effects of tibial nerve stimulation. According to the findings, PTNS in conjunction with hormone priming boosted the rats’ long-term sexual receptivity and short-term sexual drive (101).

#### 4.2.3 PNM in sexual dysfunction

A protracted pudendal nerve stimulation can cause sustained increases in vaginal blood perfusion in female rats, according to an animal experiment conducted by Rice et al. This study suggests that PNM could be utilized to treat FSD by increasing genital arousal (102). In addition, after pudendal nerve stimulation in spinally intact and spinalized rats, Bottorff et al. observed an increase in vulvar, anal, and inner thigh blood perfusion, suggesting the potential of PNM as a treatment for women with sexual dysfunction (103).

### 4.3 Use of neuromodulation in neurogenic bladder

Storage and voiding symptoms, or a mixture of the two, are included in neurogenic lower urinary tract dysfunctions (nLUTD). These conditions can be subdivided into three categories: injury/trauma [i.e., spinal cord injury (SCI), cerebrovascular injury, and pelvic surgeries], degenerative [i.e., multiple sclerosis (MS), Parkinson’s disease (PD)], and congenital (i.e., spina bifida and cerebral palsy). Based on the severity and location of their neural lesions, these neurologic patients exhibit a broad range of bladder diseases. Moreover, bowel or sexual dysfunctions may coexist with bladder problems (104).

Limited evidence supports many of the bladder management strategies used in these frequently challenging-to-treat patients, including anticholinergic medications, beta-3-adrenergic receptor agonists, injections of botulinum toxin A, intermittent catheterization, augmentation cystoplasty, and urinary diversion (105). Given that, at least 40% of neurologic patients have long-term dissatisfaction with their treatment plan (106–108), an extensive search has been conducted to determine more therapeutic options.

The incidence of nLUTD in individuals with MS is high, with a pooled prevalence of 68.41% using self-report measures and 63.95% using urodynamic examinations (109). Lower urinary tract symptoms and fecal incontinence, for instance, had significant prevalence rates (75% and 29%, respectively) in a population-based research of MS patients (110).

Multiple sclerosis patients are frequently evaluated with magnetic resonance imaging (MRI) in order to receive the best possible treatment recommendations (111, 112). Similarly, MRI is also used as surveillance in many patients with chronic SCI, which represents another group with a high prevalence of neurogenic LUTD (113, 114). A patient group that has frequently been viewed as contraindication due to the requirement for routine MRI investigations will now have greater access attributable to the introduction of the new full-body magnetic resonance imaging (MRI)-safe SNM devices (115).

#### 4.3.1 SNM in neurogenic bladder

According to a meta-analysis by Kessler et al., SNM showed encouraging outcomes in patients with neurogenic LUTD; the pooled success rate for test SNM was 68%, whereas the success rate for permanent SNM was 92% (116).

Also, as shown by Rahnama’i et al., SNM provides significant improvement in QoL, bladder symptoms, and the number of CICs in MS patients (117). Dividing neurogenic LUTD into the three subgroups [neurogenic detrusor overactivity (nDO)], neurogenic non-obstructive urinary retention, or a combination of both) revealed test success rates of 61%, 52%, and 69%, respectively (118).

A total of 47 studies were included in a systemic literature review, reporting the effectiveness of SNM in patients with neurogenic LUTD (neurogenic detrusor overactivity, non-obstructive urinary retention, or a combination of both). The pooled success rate of SNM test stimulation was 66.2% in 21 studies with a total of 887 individuals and 84.2% in 24 studies involving 428 patients with a permanent SNM (118).

Urinary retention improved at a rate of 61.5% when SNM was administered to 32 patients who had voiding dysfunction following spinal cord surgery (119). SNM proved beneficial for 62 patients who had persistent urine retention brought on by different neurological illnesses; patients’ mean maximum urinary flow rates increased and their mean postvoid residual volumes decreased significantly. The mean number of micturition, incontinence, urine urgency, and nocturia episodes was significantly lower, according to the voiding diaries. UDS revealed a decrease in maximum intravesical pressure and an increase in maximum cystometric capacity. Over an average follow-up of 4.3 years, 75.7% of the patients continued to experience the benefits of SNM (120).

SNM produces favorable results in non-obstructive urinary retention with DO and DSD in MS cases, but it has low success rates in those with non-obstructive urinary retention with an acontractile or hypocontractile bladder (121).

Furthermore, a mean follow-up of 46.8 months was found to have resulted in success rates of 73%, 62.5%, and 53% for the patients with idiopathic retention, Fowler’s syndrome, and non-Fowler idiopathic retention, respectively (>50% improvement in at least one voiding diary parameter) (122).

A 69% success rate was achieved in patients with neurogenic lower urinary tract symptoms (LUTS) and incomplete SCI after SNM implant. It was determined that there was a significant decrease in the number of catheterizations and a significant increase in the void frequency and void volume in these patients (123).

#### 4.3.2 PTNS in neurogenic bladder

When comparing the PTNS group with the control group, all voiding parameters significantly improved. There was also a substantial decrease in the number of overnight frequencies, urgency, and urgency incontinence during posttreatment episodes (P < 0.01). Additionally, compared with the control group, there was a significant drop in the posttreatment OAB symptoms scores. All urodynamic parameters (bladder capacity and compliance, detrusor overactivity (DO), maximal flow rate, and post-void residual volume) showed a statistically significant improvement in the PTNS group when compared with the control group. They came to the conclusion that PTNS is a better course of treatment than only pelvic floor muscle training for male MS patients with neurogenic-OAB symptoms (124).

PTNS decreases DO in the MS group, according to UDSs. There were 12 sessions of PTNS treatment that resulted in a significant reduction in nocturia, daytime frequency, and mean postmicturition residual volume in MS patients who were not sensitive to anticholinergic medication, according to a multicentric randomized study. The mean voided volume and QoL scores of these individuals also showed a notable improvement (117).

Studies are limited to showing the effectiveness of PTNS in this patient group. In a study including 39 patients with non-obstructive urinary retention, 59% of the patients wanted to continue the treatment, and a significant improvement was achieved in 41% according to the parameters recorded in the voiding diary (125).

#### 4.3.3 PNM in neurogenic bladder

There are limited studies to show the effectiveness of PNM in patients with neurogenic bladders. There were 15 patients with urge incontinence due to neurogenic bladders who were included in one of the first studies of PNM. The authors noted an improvement from seven incontinence episodes daily to 2.6 (27). Neurogenic urinary retention due to cerebral palsy has been effectively treated with PNM, according to a case study (126).

Based on animal research, electrical stimulation of pudendal afferents can activate the bladder and shows potential treatment for neurological disorders or injuries that affect bladder emptying (127). Hokanson et al. concluded from an animal study that stimulation of the sensory pudendal nerve led to large increases in bladder capacity, which also decreased voiding efficiency, suggesting a link between promotion of bladder filling and inhibition of voiding (128).

### 4.4 Use of neuromodulation in children

Dysfunctional voiding, nLUTD, and OAB can all lead to pediatric LUTD. The prevalence of LUTD in children ranges from 1% to 20%, resulting in a substantial economic and psychological burden on patients and their families. Neuromodulation could be of value in those patients in whom previous conventional and pharmacological therapies failed. As most of the neuromodulation techniques are invasive, they are less applicable to children. PTNS is a minimally invasive method used to treat refractory LUTDs (129).

#### 4.4.1 SNM in children

A total of 30 patients were enrolled in the study by Mason et al. on SNM implants in children with bowel bladder dysfunction. Patients had significant improvement in the QOL and symptom scores, which persisted at the median follow-up of 14.8 months. Patients who had uninhibited detrusor contractions on preoperative urodynamic assessment showed significantly greater improvements in symptoms. Of the patients, 23% had a complication: children had a high rate of lead breakage requiring operative revision, which was seen after minor trauma in those with a lower body mass index (130).

In a prospective randomized control study reported by Guys et al., 42 children with spina bifida as the primary underlying etiology were included. The patients were randomized into two groups: the SNM implant group and the conventional control group. One patient who had implantation had complete cessation of urine leakage, although periodic catheterization was still necessary. There were no significant differences in the urodynamic measures, except for functional bladder capacity, which was better in the control group, and leak point pressure, which was better in the implant group (*p* < 0.05). Interindividual differences in the implant group showed a considerable increase in compliance and functional bladder capacity at 6 and 9 months, but not at 12 months. Within the implant group, nine patients reported improved intestinal transit, five reported a complete resolution of their urine infection, and six observed ongoing bladder fullness. There was no improvement noted by any patient in the control group (131).

Haddad et al. recruited a total of 33 patients with a mean age of 12.22 years. The etiologies were mainly of neurological origin. There were 19 cases of incontinence that were mixed urine and fecal; nine cases were urinary alone; and five cases were fecal only. SNM resulted in an increase in cystometric bladder capacity but no obvious change in other urodynamic parameters. More than 75% of the respondents were positive overall, 81% in urinary function, and 78% in bowel function. SNM is superior to conservative treatment for both forms of incontinence, according to crossover analysis (*p* = 0.001). SNM is useful for treating bladder and bowel problems in children and ought to be taken into consideration prior to irreversible surgery (129, 132).

A total of 30 girls (mean age, 16 years) were included in SNM treatment for chronic constipation refractory to conservative treatment. After 3 weeks of test treatment, the mean frequency of defecation increased from 5.9 in 21 days at baseline to 17.4. In addition, the Wexner score and stomach pain dropped from 18.6 to 8.5 (*p* < 0.001) and from 3.6 to 1.5, respectively. Of the subjects, 42.9% had improvements in their symptoms over a median follow-up of 22.1 months (range, 12.2–36.8) (133). van der Wilt concluded that SNM is a therapeutic option for children with chronic constipation who do not respond to intensive oral and/or laxative therapy, providing benefits that appear to be sustained over a prolonged period of time (133).

In another study, an overall improvement in 79%–86% of children with constipation symptoms was observed, with resolution of symptoms in 40%, reduced use of an antegrade continence enema (ACE) stoma/transanal irrigation system in 12.5%–38.4%, and improvements in incontinence symptoms in 75% (134).

A total of 105 children with dysfunctional elimination syndrome whose symptoms are unresponsive to maximal medical therapy were evaluated for SNM. In 94% of cases, at least one symptom improved, but at least one symptom became worse in 11% of cases. Urinary incontinence, constipation, frequency and/or urgency, and nocturnal enuresis improved in 88%, 79%, 67%, and 66% of children, respectively, and resolved in 41%, 40%, 28%, and 28% of children, respectively, where reoperations occurred in 56%, mainly for device malfunction. It was concluded that SNM should be considered for children with dysfunctional elimination syndrome whose symptoms are refractory to maximum medical therapy, understanding that the risk of reoperation is >50% (135).

#### 4.4.2 PTNS in children

In 2015, two sham-controlled RCTs were published on the efficacy of transcutaneous tibial nerve stimulation (TTNS), which is not painful but may be better tolerated than percutaneous tibial nerve stimulation in children with OAB. Patidar et al. reported a cure rate of 66.66% and an improvement rate of 23.81% in the TTNS group (*n* = 21) compared with 6.2% and 12.5%, respectively, in the sham group (*n* = 16) (136).

When the effectiveness of percutaneous tibial nerve stimulation for children with voiding dysfunction was examined, it was shown that children with non-neurogenic bladders demonstrated higher improvements in their LUTS than children with neurogenic bladders (78% *vs*. 14%) (137). At 1-year follow-up, the cure rate was higher in children with dysfunctional voiding than in those with OAB (71% *vs*. 41%), and it stayed the same at the 2-year evaluation. Uroflowmetry-voided volume and post-void residual urine became normal in a greater number of children with dysfunctional voiding than in those with OAB (57% *vs.* 20% and 57% *vs*. 25%) (137).

A randomized work by Souto et al. also investigated the combination of PTNS with anticholinergic medication. At 12 weeks, extended-release (ER) oxybutynin at 10 mg/day and PTNS ± oxybutynin ER (10 mg/day) showed similar efficacy. These trials have not shown any severe negative effects of PTNS in children (138). Peter et al. demonstrated, in a multicenter randomized trial, a significant improvement in children with OAB receiving PTNS with comparable effects produced by ER tolterodine (79.5% reporting cure or improvement *vs*. 54.8%, *p* = 0.01). The authors stated that PTNS is safe and offers improvements in the symptoms of OAB in children, with objective effectiveness comparable with pharmacotherapy (139).

De Gennaro et al. assessed the effects of PTNS on 23 children (4–17 years old) who had unresponsive LUTS. They found that 62.5% of the children had their cystometric bladder capacity return to normal, the continent group no longer experienced unstable contractions, and the maximum flow detrusor pressure improved (*p* = 0.009) (140). However, Hoebeke et al. conducted a prospective study on 32 patients (17 boys and 15 girls, with a mean age of 11.7 years) who underwent PTNS for refractory LUTD and observed a statistically significant increase in bladder capacity (*p* = 0.001) from 185.16 to 279.19 ml and improvements in daytime frequency in 84%, urgency in 61%, and daytime incontinence in 70% (141). Ibrahim et al. assessed 20 children with refractory OAB who had PTNS and determined that the patients’ request for continued treatment was a subjective success. Continuing to be in therapy to maintain the response was chosen by 60% of patients whose symptoms improved, while 40% of them stopped because their symptoms did not get better. The bladder capacity before and after sessions was found to differ by a highly significant amount, 184.5 ± 59.14 ml *versus* 259.5 ± 77.22 ml, respectively (142).

PTNS is a safe, minimally invasive, and feasible method for children. It is reliable and effective for non-neurogenic refractory LUTD in children. It is easily applied to improve OAB symptoms and objective urodynamic changes with negligible side effects, as it has a significant effect on the voiding frequency, uroflowmetry curve, and bladder capacity in children with non-neurogenic bladder sphincter dysfunction. The combined use of PTNS and anticholinergic agents has also been investigated. In comparison with antimuscarinics, PTNS does not appear to be as cost-effective as primary care; however, it is an excellent treatment alternative for refractory OAB or in situations where antimuscarinics are not tolerated. The limited side effects of PTNS are associated with discomfort at the needle insertion site. The need for maintenance treatment after the initial 12-week course of therapy may be limited if the patient does not improve because therapy will not be continued if there is no initial improvement.

## 5 Results

The primary outcome of the review included significant changes in the pain scale from baseline to the last available follow-up, measured using VAS, PUF, GRA, and IIEF-5, whereas RAND SF-36 and EQ-5D showed improvements in the QOL of patients. The core information regarding the review and its statistical significance is shown in **Supplementary Table S1** (attached Excel). The safety and efficacy of SNM and PTNS in refractory OAB syndrome and CPP are high. Moreover, the impact of neuromodulation techniques in other conditions such as non-obstructive urinary retention, including Fowler’s syndrome and urgency incontinence, as well as frequency–urgency syndromes, was reviewed. SNM is established for its use in functional bladder syndromes, which also offer pain improvement. PTNS shows short-term improvements, but long-term effects are challenging. Studies suggesting the significance of PNM are extremely limited.

## 6 Conclusion

Neuromodulation is the most up-to-date technique in the treatment of a variety of pelvic disorders in all genders. These techniques not only decrease the severity of the symptoms but also significantly improve the QOL of affected patients.

Nowadays, neuromodulation is still endorsed only for patients who are non-responsive to standard treatments before more invasive surgery. However, existing studies did not compare the efficacy of third- and fourth-line treatments with that of SNM, PTNS, and PNM, limiting the degree of recommendations by relevant guidelines. Each technique carries its own benefits and risks; thus, decision-making on which technique to use depends on the site of pain and other individual factors. Further studies should focus more on the long-term effects, cost-effectiveness, and quality of study design and report, thus allowing neuromodulation to be considered as a first-line treatment in managing complex pelvic pain conditions.

## Author contributions

BA: Writing – original draft, Writing – review & editing. MB: Writing – review & editing. MH: Writing – review & editing.
