# Supplementary material for: Updates in pelvic neuromodulation: the role of pelvic neuromodulation in pelvic disorders
Source: Front Urol. 2024 Mar 15;4:1329305. doi: 10.3389/fruro.2024.1329305 (PMC12327268; doi:10.3389/fruro.2024.1329305)
Supplement: Supplementary file 1 [file Image_1.pdf]

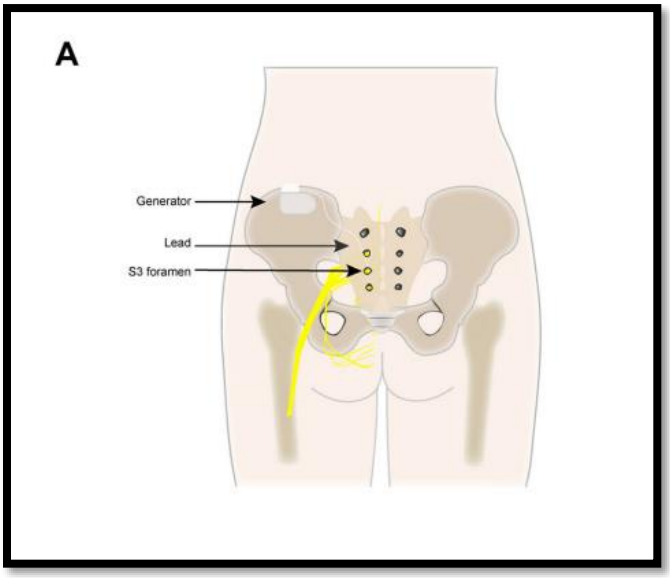

Figure 1: SNM (Sacral Neuromodulation)

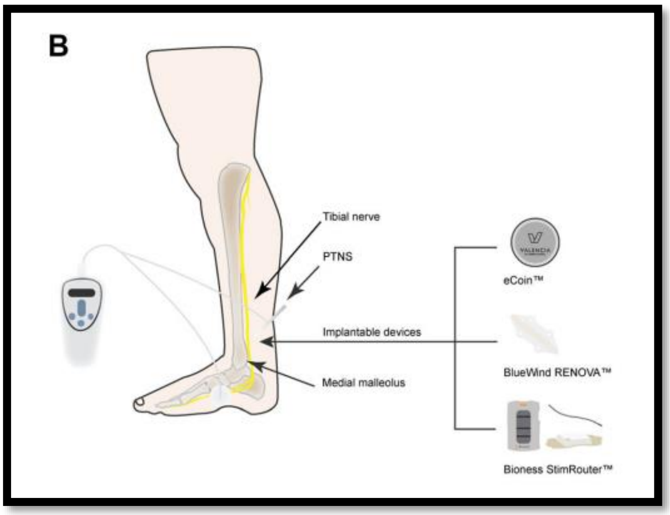

Figure 2: PTNS and Implantable PTNS (Posterior tibial nerve stimulation)

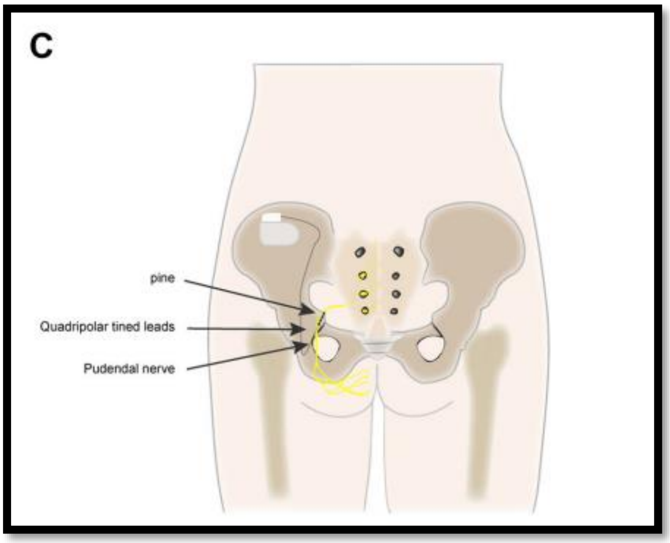

Figure 3: PNM (Pudendal nerve neuromodulation)
